# Dietary Conjugated Linoleic Acid Alters Oxidative Stability and Alleviates Plasma Cholesterol Content in Meat of Broiler Chickens

**DOI:** 10.1155/2014/949324

**Published:** 2014-10-15

**Authors:** Suriya Kumari Ramiah, Goh Yong Meng, Mahdi Ebrahimi

**Affiliations:** ^1^Institute of Tropical Agriculture, Universiti Putra Malaysia (UPM), 43300 Persiaran Serdang, Selangor, Malaysia; ^2^Faculty of Veterinary Medicine, Department of Veterinary Preclinical Science, Universiti Putra Malaysia (UPM), 43300 Persiaran Serdang, Selangor, Malaysia

## Abstract

This study was conducted to investigate the effects of dietary conjugated linoleic acid (CLA) on fatty acid composition, lipoprotein content, lipid peroxidation, and meat colour of broiler chickens. A total of 180 broiler chickens were allocated to 3 dietary treatments (0, 2.5, and 5% Lutrell) and given a standard broiler starter diet and finisher diet. Body weight of chickens and feed intake were recorded weekly. After slaughter, the breast meat was aged at 4°C for 0, 3, and 6 days. The fatty acid composition was measured in the breast meat. Body weight (BW) and feed efficiency were decreased by dietary CLA level (*P* < 0.05). Chicken fed with 2.5% Lutrell had the highest feed intake compared to the control (CON) group. The total CLA increased significantly (*P* < 0.05) in breast meat from birds supplemented with CLA. Propensity for lipid peroxidation was significantly higher after 6 days of meat storage (*P* < 0.05) and the redness in chicken breast meat was lower in CLA-fed birds (*P* < 0.05). It is also notable that a 5% Lutrell supplementation decreased the plasma total cholesterol (TC), low density protein (LDL), and HDL (high-density lipoprotein)/LDL ratio in chickens (*P* < 0.05).

## 1. Introduction

The poultry industry is constantly seeking economical methods to increase production efficiency and enhance meat quality. Poultry meat has a very high demand by consumers due to its low fat meat, low sodium, and cholesterol content [1]. To further improve its appeal to the general consumer, a variety of compounds have been incorporated in the feeding regime to enhance the health value of the poultry meat. Conjugated linoleic acids (CLA) are important ligands regulating fatty acid metabolism and deposition in animals. Prior studies have shown that CLA improved growth performance and lipid metabolism in mice and pigs [2–5], but the effect appears to be less pronounced in chickens [6, 7].

The supplementation of CLA in poultry diet has been suggested as a way to obtain meat products enriched with CLA as the level of CLA primarily in monogastric animals such as poultry and pigs is low [8]. Enrichment of chicken meat with CLA by the addition of CLA in the diet has been successfully demonstrated by several researchers [9–11]; however, the effects of CLA on meat quality and the susceptibility against peroxidative damage still remain a question.

Lipid peroxidation is a free-radical chain reaction that consists of initiation, propagation, and termination reactions [12]. Disruption of the membranes such as size, reducing processes (grinding, flaking, mincing, etc.), deboning, and cooking results in the exposure of the phospholipids to oxygen and, therefore, reduces meat quality by off-flavours, meat discolouration, drip losses, off-odour, and rancidity [12, 13]. Malondialdehyde (MDA) (a secondary product of oxidation) has been considered as an index of oxidative rancidity [14]. TBARS (thiobarbituricacid reactive substances) has been widely accepted as a sensitive assay method for lipid oxidation for assessing MDA in animal tissues [15]. Yang et al. [16] showed that CLA oxidized faster than linoleic acid (LA) because CLA has a conjugated double bond which is more vulnerable to autoxidation than a nonconjugated double bond. Study by Corino et al. [17] suggested that the decrease of peroxidation of the CLA isomers in muscles with presence of vitamin E has improved the meat quality of pigs. However, few researchers reported that CLA acts as an antioxidant in chicken meat and pork loin [18, 19]. Similarly, Chae et al. [20] reported that CLA reduced the development of lipid oxidation and extended the shelf life of beef patties.

Meat colour is an important parameter to evaluate meat quality. The rate of meat discolouration corresponds to the rate of myoglobin oxidation triggered by oxidation rancidity [21]. Du et al. [18] reported that CLA improved colour stability in fresh chicken meat. In contrast, Dugan et al. [22] reported that the lightness of pork loin was unaffected by dietary CLA. Study by Simon et al. [23] showed that fed-CLA chicken showed a decrease in plasma cholesterol concentrations. Du and Ahn [24] on the other hand found that CLA increased plasma cholesterol significantly. The level of plasma TC and LDL cholesterol correlated directly with the risk of coronary heart disease, while that of HDL cholesterol has an inverse relationship with risks of coronary heart disease [25]. Whether CLA could function as an antioxidant is still controversial, as few researchers reported that CLA is highly susceptible to oxidation [26, 27]. It thus appears that there is a gap of information pointing to the possible antioxidant properties of CLA. Therefore, this study was designed to investigate the influences of CLA supplementation on growth performance, fatty acid composition, lipid peroxidation, meat colour, and plasma lipids in broiler chicken.

## 2. Materials and Methods

### 2.1. Animal Welfare

This experimental protocol was undertaken following the guidelines of the Research Policy of Universiti Putra Malaysia on animal ethics.

### 2.2. Experimental Birds and Diets

A total of 180-day-old male broiler chicks (Cobb 500) were obtained from a local hatchery. Upon arrival, the chicks were individually wing-tagged, weighed, and randomly assigned into three treatment groups. The birds received a starter feed from day 1 until day 21 and finisher feed between days 22 to 42. Each treatment group had six replicates of 10 birds and were raised in 18 battery cages with wire floors. The cages were in a conventional open-sided house with cyclic temperatures (minimum 24°C; maximum 34°C). The relative humidity was between 80 and 90%. Feed and water were provided* ad libitum* and lighting was continuous. The chicks were vaccinated against Newcastle disease on day 7. The CLA used in this study was of commercial feed grade (Lutrell BSAF, SE, Ludwigshafen, Germany). The CLA mixture (Lutrell) consisted of equal parts of the cis-9, trans-11 CLA and trans-10, and cis-12 CLA isomers. Commencing from day 1, six cages of birds were assigned to one of the 3 dietary groups; (i) basal diet (5% palm oil) without CLA, (ii) basal diet with 2.5% Lutrell plus 2.5% palm oil (LCLA: low CLA), and (iii) basal diet with 5% Lutrell (HCLA: high CLA). The diets were in mash form. The composition of experimental diets was formulated to meet or exceed National Research Council (NRC) [28] recommendations. Tables 1 and 2 show the dietary chemical composition and fatty acid analysis of the experimental diets, respectively. The three experimental diets, which were isocaloric, are shown in Table 2. The average metabolizable energy content ranged from 3080 to 3150 Mcal/kg of the dry matter (DM) content, whilst the protein content was 22% (of DM) for starter and 20.5% (of DM) for the finisher diet. The crude fat content of all treatment diets was 5%. Body weight of chickens and feed intake were weighed at 0, 7, 14, 21, 28, 35, and 42 days from the beginning until end of the trial. Average daily gain (ADG) and conversion ratio were calculated. Ten birds were selected randomly from each treatment group. After slaughter, 10 samples (breast meat) from each treatment group were cut individually from the abdominal region and wrapped using aluminium foil. The breast samples were aged for 0, 3, and 6 days in a chiller at 3-4°C prior to oxidation analysis. The breast samples for meat colour analysis were vacuum-packed in a plastic bag and stored at −80°C for 6 days until the point of analysis.

### 2.3. Fatty Acid Extraction

Total fatty acids from breast meat, feed, and CLA were extracted using a chloroform: methanol (2 : 1 v/v) according to Folch et al. [29] as described by Ebrahimi et al. [30]. Briefly, fatty acids methyl esters (FAME) were prepared using 0.66 N potassium hydroxide (KOH) in methanol and 14% methanolic boron trifluoride (BF_3_) (Sigma Chemical Co., St. Louis, MO, USA).

### 2.4. Fatty Acid Analysis

Fatty acid methyl esters were separated and quantified by gas-liquid chromatography (Model 7890A, Agilent Technologies, USA) using a 100 m × 0.32 mm i.d. capillary column (SP-2560, Supelco, Inc., Bellefonte, PA, USA). High purity hydrogen gas (>99.998%) was used as the carrier gas at 40 mL/min. The injector temperature was programmed at 250°C and the detector temperature was 300°C. The column temperature program was initiated to run at 120°C, for 5 min, increased to 170°C at 2°C/min, held at 15 min, increased to 200°C at a rate of 5°C/min, and then held at 200°C for 5 min. The temperature was then ramped up to 235°C at a rate of 2°C/min and held for 10 min until the end of the analytical run. The identification of the peaks was made by comparing them to peaks eluted by fatty acid standards of equivalent chain lengths from a reference standard mix (C4-C24 methyl esters, Sigma Aldrich, Inc., St. Louis, MO, USA) and CLA standard mix (cis-9, trans-11 and trans-10, and cis-12 CLA, Sigma-Aldrich, Inc., St. Louis, MO, USA) as described by Ebrahimi et al. [31]. Peak areas were determined automatically using the Agilent gas chromatography Chemstation software (Agilent Technologies, USA). The fatty acid concentrations are expressed as percentage of the sum of total identified peaks measured in each sample.

### 2.5. Meat Colour

Meat colour was measured instrumentally using a ColourFlex system (Hunter Associates Laboratory, Reston, USA). The samples were evaluated for lightness (*L* ^*^), redness (*a* ^*^), and yellowness (*b* ^*^). The spectrocolorimeter was standardized using white (*L* ^*^ = 100) and black (*L* ^*^ = 0) standard tiles, before being used. On the day of measurement, samples in vacuum packs were placed at 4°C and allowed to bloom for 1 hour prior to colour measurement. The samples were placed directly onto the colour meter and measured. A total of three readings of the *L* ^*^, *a* ^*^, and *b* ^*^ values and spectral reflectance (400–700 nm) were collected from different sites of each sample and averaged.

### 2.6. Lipid Peroxidation

Meat samples (1 g) were homogenized in 4 mL 0.15 M KCl + 0.1 mM BHT with Ultra-Turrax homogenizer (1 min, medium speed). After homogenization, 200 *μ*L of the samples was mixed with TBARS solution and heated in a water bath at 95°C for 60 min until the development of a pink colour. After cooling, 1 mL of distilled water and 3 mL of n-butyl alcohol were added to the extracts and vortexed. The mixtures were centrifuged at 2991 G for 10 min. Absorbance of the supernatant was read against an appropriate blank at 532 nm using a spectrophotometer (Secomam, Domont, France). The TBARS values were calculated from a standard curve of 1,1,3,3-tetraethoxypropane and expressed as mg MDA/kg meat.

### 2.7. Plasma Lipids Profile

Blood sampling was carried out on ten animals from each group at the point of sacrifice. Blood samples were collected by severing the carotid artery and jugular vein at the base of the neck. The blood samples were then centrifuged at 1077 G for 10 min to collect the plasma. The plasma samples were then analyzed for TC, HDL, LDL, and triglycerides (TG), using analytical kits (Pointe Scientific Inc., MI, USA) and determined colourimetrically on a Hitachi 902 automatic chemical analyzer (Roche International, Basel, Switzerland).

### 2.8. Statistical Analysis

Results were analyzed using the analysis of variance (ANOVA) procedure, with the CLA levels as the main effect. Average daily weight gain (ADG) and conversion ratio were analyzed using the MIXED procedure of SAS with the time of recording in a repeated measure analytical model. The meat quality data (colour value and TBARS value) was analyzed in a 3 × 3 factorial design (3 levels of diets × 3 levels of postmortem aging periods). Fatty acid, plasma TC, TG, HDL, LDL, and ratio of HDL/LDL data were analyzed using the one-way ANOVA, using the MIXED procedure of the SAS software package, version 9.1 (SAS Institute Inc., Cary, NC). Differences of *P* < 0.05 were considered to be significant. Prior to analysis, all the data was checked for conformance to normality using the PROC UNIVARIATE procedure of the SAS software. All the results in the tables are presented as means ± standard error of the means.

## 3. Results

### 3.1. Fatty Acid Composition of Experimental Diets

#### 3.1.1. Growth Performances

The overall effect of CLA feeding on growth performance of broiler chicken is depicted in Table 3. It is evident that the body weight gain was reduced by increasing level of dietary CLA (*P* < 0.05) in comparison to the CON group. However, the body weight of birds from the CON and LCLA dietary groups was not significantly (*P* > 0.05) different. Higher feed intake was noted among birds supplemented with CLA compared to the CON group across the whole experimental period. The feed intake was not significantly (*P* > 0.05) different among all the treatment groups during starter and finisher phase. Between 1 and 42 days, birds fed with LCLA diet showed significantly higher feed intake compared to CON (*P* < 0.05). The feed efficacy of birds in all treatment groups was found to be significantly different at starter phase, finisher phase, and throughout the trial period. As a result, the feed efficiency ratio was significantly (*P* < 0.05) increased from 1.47 to 1.83 with the increasing level of dietary CLA.

#### 3.1.2. Breast Meat Fatty Acids

Generally, fatty acid composition of breast meat was affected significantly by the level of CLA concentrations in the diet (Table 4). Between the two major CLA isomers, trans-10 and cis-12, CLA was incorporated at a higher level than the cis-9 and trans-11 CLA. It is also noteworthy to point out that no CLA isomers were detected in the CON group. In contrast, the CLA content increased significantly (*P* < 0.05) with the increasing doses of CLA, from none detected in the CON group to 134.09 mg/100 g (LCLA) and 188.54 mg/100 g (HCLA). C16:1 n-7 was not affected by the addition of CLA in the diet (*P* < 0.01). However, 18:1 n-9 and the total MUFA percentages were reduced (*P* < 0.05) in HCLA fed chickens. Dietary CLA supplementation has no significant effect on all individual SFA compared to the CON group (*P* > 0.05). A reduction of n-6 PUFA (*P* < 0.05) was attributed to the reduction of C18:2 n-6 and C20:4n-6 in HCLA groups when comparisons were made to the CON birds. The relative concentration of total polyunsaturated fatty acids (PUFA) in the meat was also not significantly (*P* > 0.05) different between broilers fed CLA and non-CLA dietary groups. In fact, the n-3 PUFA, n-6: n-3 fatty acid ratios (FAR), and PUFA: SFA in the meat were similar (*P* > 0.05) across the treatment groups.

### 3.2. Lipid Peroxidation

**Figure 1:**
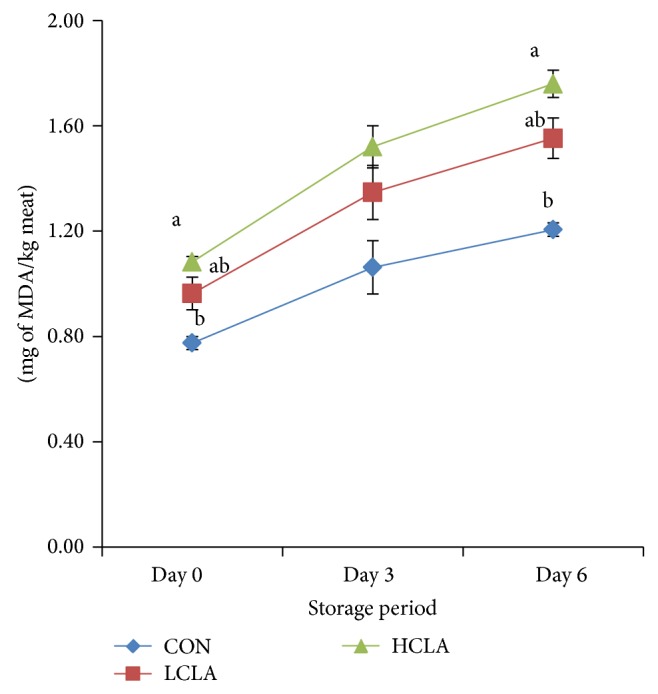
TBARS values (mg/kg) of chicken breast meat from different treatment after 0, 3, and 6 days of storage. CON: control; LCLA: low conjugated linoleic acid; HCLA: high conjugated linoleic acid. Data presented as means with pooled SEM (standard error of the mean) (*n* = 6/per treatment group).  ^a,b^Means with no common superscript at the same storage period differ significantly (*P* < 0.05).

The TBARS values in CON, LCLA, and HCLA dietary groups were expressed as micrograms of malondialdehyde (MDA) per kilogram of meat (Figure 1). In general, TBARS values in CON meat were low, but the CLA supplemented groups have significantly (*P* < 0.05) higher TBARS values compared to the unsupplemented CON group. The lipid oxidation in CLA dietary groups was merely onefold higher, at day 0 and day 6 compared to the CON group. No significant (*P* > 0.05) difference was noted in all treatment groups after 3 days of aging. The TBARS value was also found to be increasing with the increasing dose of CLA in the diet and also increased in tandem with the increasing storage period of the meat.

### 3.3. Meat Colour

Results for meat colour are shown in Table 5. After 6 days of storage, it was noted that adding CLA in the diet did not influence *L* ^*^ values in chicken meat. Higher lightness (*L* ^*^) values were observed with an increase of CLA dosage, but these changes were not significantly different (*P* > 0.05) compared to the CON group. However, the redness (*a* ^*^) values in the CLA treated groups were significantly (*P* < 0.05) lower compared to CON. Yellowness (*b* ^*^) values were significantly (*P* < 0.05) higher among meats from HCLA birds than the LCLA group.

### 3.4. Plasma Lipids Profile

The effects of CLA on plasma TC, TG, LDL, HDL, and ratio of HDL/LDL for chicken are reported in Table 6. The plasma TC, LDL, HDL, and the ratio of HDL/LDL in the HCLA group were significantly lower (*P* < 0.05) compared to both LCLA and CON groups. There were no significant (*P* > 0.05) differences noted for plasma TC, LDL, HDL, and the ratio of HDL/LDL between LCLA and CON groups. The TG concentrations were the same across all treatment groups (*P* > 0.05).

## 4. Discussion

### 4.1. Growth Performances

The CLA preparation used in this study contained equal amounts of the cis-9, trans-11 CLA and the trans-10, and cis-12 CLA. This is a crucial part of the experiment as it has been suggested that the biological action of the cis-9, trans-11 CLA and the trans-10, and cis-12 CLA might be different [32]. It is postulated that the effects of CLA presented in this study could therefore result from either or both of these isomers. Previously, Szymczyk et al. [33] and Suksombat et al. [7] showed that dietary CLA at levels of 0.0, 0.5, 1.0, and 1.5% in the diets reduced growth performances in broilers, and this was consistent with the present data. However Zhang et al. [34] investigated the effects of increasing CLA concentration at 0, 2.5, 5.0, or 10.0 g pure CLA/kg for 6 weeks in broiler chickens and observed no alteration on the growth performances. One possible explanation behind the reduction in weight gain associated with CLA supplementation may be attributed to the overall increase in metabolic rate of birds, stemming from the increase in fatty acid oxidation as a result of CLA supplementation as proposed earlier by other workers [3]. However, the net effect of these increases in metabolic rate may not necessarily trigger the enhancements in growth performance [5, 35], and hence the reduction of weight gain as observed in the current study. Our finding with broilers was not entirely in agreement with earlier observations that CLA depressed feed intake in poultry [36–38], pigs [4], and mice [3]. However, our current findings were consistent with the findings by Zhang et al. [34], who also reported higher conversion ratio in the CLA supplemented group.

### 4.2. Fatty Acid Composition of Breast Meat

The majority of studies showed that CLA feeding leads to an increasing proportion of saturated fatty acids (SFA), while decreasing monounsaturated fatty acids (MUFA) in broiler chickens [9, 10, 33]. Our results pointed to the possible inhibitory action/actions of CLA isomers on delta-9-desaturase activity [39], thus explaining the observed low proportion of MUFAs especially C18:1n-9 in birds in the HCLA group that was fed with higher amounts of CLA.

Our data also showed that dietary HCLA reduced the accumulation of linoleic acid (LA: C18:2n-6) and arachidonic acid (ARA: 20:4n-6) in meat. This is in agreement with previous findings by Sirri et al. [10] and Zlatanos et al. (2008) [40]. According to Hansen Petrik et al. [41], CLA acts as substrate for delta-6-desaturase that inhibits the conversion of linoleic acid to ARA. Furthermore, the inhibition of delta-6-desaturase in CLA dietary was also consistent with our results where the n-3 PUFA concentrations in the meats were not modified by the CLA dietary supplementation. In fact, this claim is further substantiated by claims from Zlatanos et al. [40] who suggested that CLA appears to act as an antagonist against n-6 fatty acids. In the present study, CLA did not affect the level of PUFA, which was consistent with earlier work done [41–43]. Nevertheless, other authors have also found the opposite trend of the effect of CLA on PUFA [44–46]. The plausible explanation is that the ability of CLA to alter PUFA levels is very much tissue and species dependent [47].

The CLA was expected to be incorporated into chicken breast meat because monogastric animals tend to deposit fatty acids in their tissue in the form in which they are consumed [48]. In this study, although diets contained equal concentration of both isomers, we found that the deposition of trans-10, cis-12 CLA in chicken breast meat was higher than the cis-9, trans-11 CLA isomer. In this regard, dietary CLA supplementation caused a dose-dependent incorporation of CLA in chicken tissues. In contrast, many reports indicated that cis-9, trans-11 CLA was deposited predominantly into meat compared to trans-10, cis-12 CLA. This phenomenon seems to be dependent on the amount of different isomers in CLA supplementation [23, 46]. Based on our data, trans-10, cis-12 CLA was incorporated efficiently into chicken breast meat and may have been subjected to lesser degree of metabolic modifications during the incorporation compared to cis-9, trans-11 CLA. This may support the theory that the trans-1, cis-12 CLA isomer is more efficiently driven through peroxidation in cells of the meats, kidneys, adipose tissue, or liver than the cis-9, trans-11 isomers [49]. The total CLA concentration in meats was the highest among animals from the HCLA, followed by LCLA and CON groups. The importance of CLA in the human diet is well established and shown to have many cancer-fighting, antiobesity, and antiatherosclerotic properties [50]. The main sources of CLA for humans are through the consumption of milk, milk products, and other animal-derived products. By feeding CLA supplemented diets to broilers [6, 9], the CLA content of poultry meat may be increased by 40 times in the breast and thigh meat. Thus CLA-enriched poultry is an attractive option to increase the intake of CLA in human populations. However, despite the high concentration of CLA in breast and thigh meat, this may only account for about 10% of the daily recommended intake of CLA in humans which is estimated to be 5 g/day [51].

### 4.3. Lipid Peroxidation

In terms of lipid peroxidation, our result is in contrast with that of Du et al. [18], who found that dietary CLA resulted in a decrease of TBARS values of chicken meat patties after storage under aerobic conditions. On the same note, Joo et al. [52] proposed that dietary CLA reduced TBARS levels and lipid oxidation of pork loin. The current data are in agreement with Banni et al. [27] who reported that CLA does not possess antioxidant properties. In fact, Yang et al. [16] reported that CLA was very susceptible to autooxidation when exposed to air. Du et al. [18] reported that the proportional increase of aldehydes in CLA meat during 6-day storage period indicated that CLA has no antioxidant properties. Another report by Leung and Liu [53] stated that CLA may not possess antioxidant properties due to the chemically reactive nature of alkene bonds in trans-10, cis-12 CLA and cis-9, and trans-11 CLA molecules. Cis-9, trans-11 CLA possesses strong prooxidant properties while prooxidant activity was not observed in trans-10, cis-12 CLA [53]. Rendering further support to the above statement, the rapidity of CLA oxidation was probably due to the formation of the unstable free-radical intermediate [54]. In test tube studies, CLA was found not to act as an antioxidant but to accelerate lipid peroxidation instead [55]. This may partly explain the high TBARS value in our study.

### 4.4. Meat Colour

The rate of meat colour discolouration is related to lipid oxidation [56]. Generally, if the dietary CLA supplementation was effective as an antioxidant, meat oxidation, rancidity, and ultimately the degradation of meat colour pigment will be inhibited during storage [52]. Contrary to our results Joo et al. [52] reported that 5% dietary CLA showed significantly lowered *L* ^*^ values than the CON in pork. In our study, the *A* ^*^ (redness) values decreased in chicken breast meat supplemented with LCLA and HCLA compared to CON group. This may be attributed to myoglobin oxidation which is associated with oxygen content at the point of storage [57]. Our findings indicated that the higher *b* ^*^ value observed in HCLA could be due to increased oxidation with time [58]. Overall, our results are in agreement with Wiegand et al. [48] who observed that meat from CLA-fed pigs had higher *b* ^*^ (yellowness) values, leading to a more yellowish colouration. On the other hand, there are few reports which proposed that CLA had negligible effects on meat colour indices in chicken [24, 59]. However, these discussions should take into account the variability of antioxidant versus prooxidative effects of CLA across all studies reviewed so far.

### 4.5. Plasma Lipids Profile

The plasma TG was not generally expected to differ across groups, as per findings of Lee et al. [60] and Nicolosi et al. [61]. Stangl [62] reported that CLA mixtures of 3% and 5% in the diets reduced the LDL and HDL cholesterol in rats. Similarly CLA-fed rabbits demonstrated significant reduction in LDL/HDL and TC/HDL cholesterol ratios [60]. Our study indicated that 5% dietary CLA affected cholesterol levels in broiler chicken. This suggested that the changes of plasma lipoproteins may most probably be due to trans-10, cis-12 CLA, as pointed out earlier by de Deckere et al. [63]. de Deckere et al. [63] suggested that trans-10, cis-12 CLA isomer is also responsible for decreasing the fasting values of LDL and HDL cholesterol, increasing very low-density lipoprotein- (VLDL-) triacylglycerol, and reducing epididymal fat pad weights, whereas cis-9, trans-11 CLA isomer demonstrated negligible effects on the same parameters in the hamster model. There are evidences that trans-10, cis-12 CLA reduced lipoprotein lipase activity, but cis-9, trans-11 in contrast did not affect these biochemical activities in cultured 3T3-L1 adipocytes [64].

## 5. Conclusions

Based on the results, it can be concluded that chicken supplemented with dietary CLA exhibited lower body weight and feed efficiency. The CLA in meat increased substantially with CLA feeding dose and concurrently this resulted in increase of TBARS values in chicken meat. Higher lipid oxidation in turn could potentially lead to meat spoilage. The current study also found no evidence in favour of CLA as an antioxidant; instead CLA is more likely to precipitate oxidation itself. CLA feeding also resulted in the reduction of plasma TC, LDL, and the ratio of HDL/LDL, particularly among the HCLA chickens. It is thus concluded that the incorporation of CLA isomers into chicken meat should be considered as a viable option to increase CLA intake in human populations.

## Figures and Tables

**Table 1:** Ingredients and chemical compositions of experimental diets.

Ingredient (% DM)	Experimental diets					
	Starter (1–21 days)			Finisher (22–42 days)		
	CON	LCLA	HCLA	CON	LCLA	HCLA
Corn	51.17	51.17	51.17	58.9	58.9	58.9
Soybean meal	40.56	40.56	40.56	32.22	32.22	32.22
Palm oil	5	2.5	—	5	2.5	—
Lutrell	—	2.5	5	—	2.5	5
DiCalcalcium phosphate	1.34	1.34	1.34	1.4	1.4	1.4
Limestone	1.7	1.7	1.7	1.19	1.19	1.19
Common salt	0.4	0.4	0.4	0.4	0.4	0.4
^ 1^Vitamin premix	0.05	0.05	0.05	0.05	0.05	0.05
^ 1^Mineral premix	0.05	0.05	0.05	0.05	0.05	0.05
DL-methionine	0.26	0.26	0.26	0.3	0.3	0.3
Lysine	—	—	—	0.5	0.5	0.5
^ 2^Chemical composition						
Crude protein (% DM)	22.00	22.00	22.00	20.5	20.5	20.5
Metabolizable energy (Kcal/kg)	3080	3080	3080	3150	3150	3150
Crude fat (%)	7.27	7.27	7.27	7.50	7.50	7.50
Phosphorus (% DM)	0.45	0.45	0.45	0.42	0.42	0.42
Calcium (% DM)	1.00	1.00	1.00	0.9	0.9	0.9
Methionine (% DM)	0.55	0.55	0.55	0.5	0.5	0.5
Lysine (% DM)	1.20	1.20	1.20	1	1	1
Na (% DM)	0.20	0.20	0.20	0.15	0.15	0.15

CON: CON; LCLA: low conjugated linoleic acid; HCLA: high conjugated linoleic acid.

^1^Premixes contributed the following nutrients per kilogram of complete feed: vitamin A, 2300 IU; vitamin D_3_, 400 IU; vitamin E, 1.8 mg; vitamin B_12_, 3.5 mg; riboflavin, 1.4 mg; pantothenic acid, 2 mg; nicotinic acid, 7 mg; pyridoxine, 0.25 mg; folic acid, 0.15 mg; menadione, 0.3 mg; thiamin, 0.15 mg; manganese oxide, 35 mg; ferrous sulfate 35 mg; zinc oxide, 30 mg; copper sulfate, 60 mg; cobalt carbonate, 5 mg; potassium iodine, 0.6 mg; selenium vanadate, 0.09 mg. CLA used in this study was Lutrell pure, BASF, Germany, which contained 60% CLA. Dietary inclusion of 5% and 2.5% Lutrell will be 1.5% and 3% of both CLA isomers, respectively.

^2^Calculated values.

**Table 2:** Fatty acid composition of experimental diets.

Fatty acids	Experimental diets					
	Starter (1–21 days)			Finisher (22–42 days)		
	CON	LCLA	HCLA	CON	LCLA	HCLA
C12:0	0.73	0.48	0.23	0.76	0.51	0.26
C14:0	3.19	1.69	0.19	3.23	1.72	0.22
C16:0	23.00	15.72	8.43	23.34	16.06	8.78
C16:1n-7	0.53	0.28	0.03	0.53	0.28	0.03
C18:0	2.71	14.52	26.32	2.81	14.62	26.42
C18:1n-9	29.74	23.53	17.30	31.33	25.12	18.90
C18:2n-6	12.00	10.05	8.03	12.18	10.20	8.20
C18:3n-3	0.79	0.54	0.29	0.82	0.57	0.32
cis-9, trans-11CLA	0.00	2.97	5.97	0.00	3.00	5.95
trans-10, cis-12CLA	0.00	2.92	5.90	0.00	2.93	5.91
^ a^Total SFA	29.63	32.41	35.18	30.13	32.91	35.69
^ b^Total MUFA	30.27	23.81	17.34	31.86	25.40	18.94
^ c^Total n-3PUFA	0.79	0.54	0.29	0.82	0.57	0.32
^ d^Total n-6PUFA	12.00	10.05	8.03	12.18	10.20	8.20
^ e^Total PUFA	12.79	10.59	8.33	13.00	10.77	8.52
^ f^n-6 : n-3FAR	15.16	18.54	27.53	14.77	17.75	25.27
PUFA : SFA	0.43	0.33	0.24	0.43	0.33	0.24
^ g^Total CLA	0.00	5.89	11.86	0.00	5.93	11.86

CON: control; LCLA: low conjugated linoleic acid; HCLA: high conjugated linoleic acid.

The data are expressed as the g/kg feed.

^a^Total SFA = sum C12:0 + C14:0 + C16:0 + C18:0.

^b^Total MUFA = sum of C16:1 + C18:1n-9.

^cd^n-3 PUFA = sum of C18:3n-3.

^d^n-6 PUFA = sum of C18:2n-6.

^e^Total PUFA = n-3 PUFA + n-6 PUFA.

^f^n-6 : n-3 FAR (fatty acid ratio) = sum of (C18:2n-6 ) ÷ sum of (C18:3n-3).

^g^Total CLA = sum of cis-9, trans-11 CLA + trans-10, cis-12CLA.

**Table 3:** Body weight, feed intake, and gain/feed from broiler chickens fed with CON, LCLA, and HCLA.

Parameters	Experimental diets			SEM	*P* value
	CON	LCLA	HCLA		
Body weight (BW) (g)					
Days 0–21	644.9^a^	593.3^b^	569.7^c^	5.5	<0.0001
Days 21–42	1479.6^a^	1466.1^a^	1405.0^b^	11.9	0.0205
Days 1–42	2120.6^a^	2077.9^a^	1974.7^b^	13.6	<0.0001

Average daily gain (ADG) (g)					
Days 0–21	30.711^a^	28.492^b^	27.120^c^	9.966	<0.0001
Days 22–42	69.811^a^	70.451^a^	66.900^b^	12.323	0.0202
Days 1–42	50.492^a^	49.471^a^	47.010^b^	16.115	<0.0001

Feed intake (g)					
Days 0–21	943.0	974.0	1008.2	21.4	0.379
Days 21–42	2428.8	2668.0	2584.7	50.8	0.180
Days 1–42	3371.8^b^	3642.1^a^	3592.9^ab^	52.3	0.015

Gain/feed (G : F)					
Days 0–21	0.685^a^	0.610^b^	0.564^c^	0.006	<0.0001
Days 21–42	0.595^a^	0.567^b^	0.544^c^	0.004	<0.0001
Days 1–42	0.630^a^	0.572^b^	0.549^c^	0.004	<0.0001

CON: control; LCLA: low conjugated linoleic acid; HCLA: high conjugated linoleic acid. Data presented as means with pooled SEM (*n* = 60/treatment). ^a,b,c^Means within a row with no common superscript differ significantly (*P* < 0.05).

**Table 4:** Fatty acid composition of breast meat (mg/100 g meat) of broiler chicken fed with different inclusion levels of CLA in the diet.

Treatments	Experimental diets			SEM	*P* value
	CON	LCLA	HCLA		
C12:0	7.50	10.31	7.84	1.95	0.060
C14:0	72.36	54.41	59.50	9.66	0.231
C16:0	586.85	491.84	433.56	21.95	0.763
C16:1n-7	113.98	88.38	52.46	18.69	0.398
C17:0	18.88	22.62	26.93	4.10	0.160
C18:0	323.55	305.25	293.06	14.66	0.758
C18:1n-9	683.17^a^	611.39^a^	536.97^b^	26.87	0.031
C18:2n-6	438.50^a^	321.93^ab^	280.90^b^	13.21	<0.0001
C18:3n-3	13.31	19.54	14.07	1.32	0.077
cis-9, tran-11 CLA	0.00^c^	28.42^b^	40.80^a^	3.35	<0.0001
trans-10, cis-12 CLA	0.00^c^	105.67^b^	147.74^a^	15.11	<0.0001
C20:4n-6	98.98^a^	93.24^a^	78.99^b^	10.87	0.034
C20:5n-3	10.65	21.98	11.26	2.40	0.090
C22:5n-3	22.26	21.98	23.12	3.92	0.868
C22:6n-3	23.72	22.84	11.26	5.34	0.094
^ a^Total SFA	1009.14	884.43	820.88	28.40	0.814
^ b^Total MUFA	797.15^a^	699.76^ab^	589.44^b^	33.97	0.020
^ c^Total n-3PUFA	69.94	86.33	59.70	10.28	0.118
^ d^Total n-6PUFA	537.48^a^	415.17^ab^	359.89^b^	15.85	0.015
^ e^Total PUFA	607.42	501.50	419.59	18.33	0.506
^ f^n-6 : n-3 FAR	7.69	4.81	6.03	0.75	0.112
PUFA : SFA	0.60	0.57	0.51	0.04	0.612
^ g^Total CLA	0.00^c^	134.09^b^	188.54^a^	17.23	<0.0001

CON: control; LCLA: low conjugated linoleic acid; HCLA: high conjugated linoleic acid.

The data are expressed as mg/100 g meat. Data presented as means with pooled SEM (*n* = 10/treatment). ^a,b^Means within a row with no common superscript differ significantly at *P* < 0.05.

^a^Total SFA = sum C12:0 + C14:0 + C16:0 + C18:0.

^b^Total MUFA = sum of C16:1n-7 + C18:1n-9.

^c^n-3 PUFA = sum of C18:3n-3 + C20:5n-3 + C22:5n-3 + C22:6n-3.

^d^n-6PUFA = sum of 18:2n-6 + C20:4n-6.

^e^Total PUFA= n-3 PUFA+ n-6 PUFA.

^f^n-6 : n-3 FAR (fatty acid ratio) = sum of (C18:2n-6 + C20:4n-6) ÷ sum of (C18:3n-3 + C20:5n-3 + C22:5n-3 + C22:6n-3).

^g^Total CLA = sum of cis-9, trans-11 CLA + trans-10, cis-12 CLA.

**Table 5:** Colour values of chicken breast meat from different treatments.

Treatments	Experimental diets			SEM	*P* value
	CON	LCLA	HCLA		
*L* ^*^	51.93	55.65	55.49	0.792	0.071
*a* ^*^	11.129^a^	7.230^b^	7.676^b^	0.573	0.003
*b* ^*^	20.285^a^	16.847^b^	19.114^a^	0.481	0.014

*L* ^*^: lightness; *a* ^*^: redness; *b* ^*^: yellowness; CON: control; LCLA: low conjugated linoleic acid; HCLA: high conjugated linoleic acid. Data presented as means with pooled SEM (standard error of the means) (*n* = 10/per treatment group). ^a,b^Means within a row with no common superscript differ significantly (*P* < 0.05).

**Table 6:** The effects of CLA on cholesterol, triglycerides, and lipoproteins (mmol/L) indices in broiler chickens.

Parameter	CON	LCLA	HCLA	SEM	*P* value
^ 1^TC	3.255^a^	3.092^a^	2.456^b^	0.044	0.0034
^ 2^TG^ns^	0.375	0.380	0.310	0.020	0.567
^ 3^HDL	2.397^a^	2.227^a^	1.840^b^	0.043	0.0064
^ 4^LDL	0.552^a^	0.577^a^	0.358^b^	0.021	0.022
HDL/LDL	2.924^a^	3.032^a^	2.272^b^	0.057	<0.0001

^1^TC: total cholesterol; ^2^TG: triglycerides; ^3^HDL: high-density lipoprotein; ^4^LDL: low-density lipoprotein; CON: control; LCLA: low conjugated linoleic acid; HCLA: high conjugated linoleic acid. Data presented as means with pooled SEM (standard error of the means) (*n* = 10 per treatment group).^ a,b^Means within row with no common superscript differ significantly at (*P* < 0.05). ^ns^no significant different.
